# Improving the Diagnostic Information Recorded in the Electronic Case Records in a Mental Health Service: A Quality Improvement Project

**DOI:** 10.1192/bjo.2025.10344

**Published:** 2025-06-20

**Authors:** Matilda Burton, Nilamadhab Kar, Annabel Ariyathurai, Mohamed Yaasir Mohamudbucus, Vijay Perla

**Affiliations:** Department of Psychiatry, Black Country Healthcare NHS Foundation Trust, Wolverhampton, United Kingdom

## Abstract

**Aims:** Up-to-date and accurate diagnostic information is essential for many reasons including effective clinical management; however, this may not be the case for psychiatric patients seen across different healthcare settings. It was intended to explore the documentation of both psychiatric and physical diagnoses within the electronic case records (ECR), and their quality based on the character of International Classification of Diseases coding; and to update the information where appropriate.

**Methods:** Records of 114 consecutive patients attending outpatient clinics were studied. An initial audit of psychiatric and physical diagnoses was conducted; followed by updating these where additional information was available.

**Results:** The sample consisted of 65 (57.0%) female and 49 (43.0%) male patients, with a mean age of 41.3±13.6 and 37.4±12.9 years. The period in psychiatric services was less than one year in 20 (17.5%); between one and five years in 57 (50.0%) and more than five years in 37 (32.5%) patients. Comorbidity of psychiatric diagnoses was present in 39.5%; similarly, 51.8% had associated physical diagnoses, with 29.8% having more than one physical diagnosis.

Before the intervention, only 35 (30.7%) patients had psychiatric diagnoses available in the designated place in ECR, although diagnoses were available in 97.4% of cases elsewhere in the case record. In 83 (72.8%) patients additional psychiatric diagnoses could be entered. Pre- and post-project, the mean number of psychiatric diagnoses changed from 0.7±1.4 to 1.0±1.3 (p<0.001), and that for physical diagnoses were 0.3±0.9 and 1.1 ± 1.4 (p<0.001). The number of characters of ICD diagnoses also changed, such as three (1.8% v 4.4%), four (28.9% v 86.8%) and five (0.0% v 8.8%) respectively (p<0.01).

Initially, 15 (13.2%) patients had physical diagnoses; however, it was updated in 43 (37.7%) patients. In 28 (24.6%) patients physical diagnoses were taken from the GP records; the total number of diagnoses entered in this process was 55, with a mean of 0.5±1.1 per patient.

There was no difference between genders in the documentation of psychiatric or physical diagnoses initially; however, following updating, the mean number of psychiatric diagnoses for males (2.1 ± 1.6) was significantly more than for females (1.6±0.9; p<0.05).

**Conclusion:** A focused effort to review and document psychiatric and physical diagnoses appropriately can improve the quality of ECR and support patient care. This is especially relevant for patients being seen in different settings of primary and secondary care centres.

